# Broad and Fine Scale Range Shifts of a Species at Risk Across North America

**DOI:** 10.1002/ece3.71537

**Published:** 2025-06-05

**Authors:** Kelsey Freitag, Ann E. McKellar, David W. Bradley, Scott A. Flemming, Steffi LaZerte, Mateen Shaikh, Matthew W. Reudink

**Affiliations:** ^1^ Department of Biological Sciences Thompson Rivers University Kamloops British Columbia Canada; ^2^ Wildlife Research Division Environment and Climate Change Canada Saskatoon Saskatchewan Canada; ^3^ Birds Canada Delta British Columbia Canada; ^4^ Canadian Wildlife Service Environment and Climate Change Canada Delta British Columbia Canada; ^5^ Steffi LaZerte R Programming and Biological Consulting Brandon Manitoba Canada; ^6^ Department of Mathematics & Statistics Thompson Rivers University Kamloops British Columbia Canada

**Keywords:** agriculture, climate change, conservation, distribution, grassland birds, habitat loss, long‐billed curlew, *Numenius americanus*, occupancy

## Abstract

Changes to the distributions of bird populations are becoming increasingly common as climate change and habitat loss continue to alter environments at a global scale. Grassland habitats have been disproportionately impacted by these stressors, leading to unprecedented declines of grassland bird species. Many grassland birds, such as the long‐billed curlew ( *Numenius americanus* ), have wide ranges across North America, and thus may face different threats and pressures in different parts of their range. Community science databases, such as eBird provide large‐scale, long‐term temporal and spatial data, allowing for studies that examine changes in species distribution both regionally and range‐wide. Using 13 years of eBird data, we examined changes to the long‐billed curlew breeding range boundaries and centroid position in North America, and centroid position within eight Bird Conservation Regions (BCR; groupings of similar bird communities and habitats across North America) in which the species occurs. We found an overall northward range expansion of approximately 198 km. At the BCR scale, the Northern Rockies (BCR 10) also showed a northern centroid shift. The Prairie Potholes showed an eastern centroid shift, consistent with a declining population in the northeast Canadian portion of this BCR. Furthermore, we found a pattern of western centroid shifts in several BCRs, consistent with grassland loss in eastern North America. These results reinforce the importance of understanding both range‐wide and regional population dynamics to effectively manage at‐risk species.

## Graphical abstract

Climate change and habitat loss are driving distributional changes of long‐billed curlews across their North American breeding range. Although climate change appears to be influencing curlew range dynamics at a broad, range‐wide scale, at the regional level, factors such as habitat loss are shifting local distributions. These findings highlight the importance of investigating changes in distributions at multiple scales to effectively predict how climate change and habitat loss will impact vulnerable species.

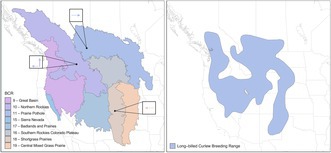

## 1 Introduction

The Earth's temperatures are rising, impacting animal populations on a global scale (Chen et al. 2011; Mac Nally et al. 2009; Mantyka‐Pringle et al. 2012, 2015; Parmesan and Yohe 2003) and shifting the distribution of many bird species poleward toward northern/southern latitudes or higher elevations (Parmesan and Yohe 2003; Hitch and Leberg 2007; Nixon et al. 2016; Rushing et al. 2020). However, these patterns are complex and often differ markedly across taxa (La Sorte and Jetz 2012; Rubenstein et al. 2023). Combined with other anthropogenic stressors such as habitat loss and degradation, bird populations across North America are facing unprecedented declines (Jaureguiberry et al. 2022; Mantyka‐Pringle et al. 2012; Rosenberg et al. 2019). Grasslands have been disproportionately impacted by land use change, with native habitat lost to agricultural conversion and urbanization (Comer et al. 2018; Vickery et al. 2000), resulting in the loss of over 60% of the native grasslands in North America (Comer et al. 2018). Furthermore, the remaining grasslands have been degraded through grazing pressure, invasive plants, and woody encroachment from fire suppression (Stanton et al. 2018; Vickery et al. 2000). Not surprisingly, grassland bird species have experienced the most drastic decline of all bird species since the 1970s (Rosenberg et al. 2019; Birds Canada and Environment and Climate Change Canada 2024).

The long‐billed curlew ( *Numenius americanus* ; hereafter “curlew”) is a large shorebird species that breeds throughout western North America in short grass and mixed grass prairies (Cannings 1999; COSEWIC 2002; Fellows and Jones 2009). Curlew populations have shown variable trends across their North American range, with a negative trend from 1980 to 2000 (Sauer et al. 2001, as reported by COSEWIC 2002) but a slight increase from 2011 to 2021 (Smith et al. 2019). Canadian trends remain highly variable, where some provinces, such as Alberta, show population increases while others, such as British Columbia, show strong population decreases (Smith et al. 2024). This variability suggests that curlew populations are being influenced by region‐specific factors, which may include habitat availability and climate pressures. Investigating these patterns is crucial for informing species‐specific conservation and understanding how similar species may respond to these stressors.

The curlew's breeding range historically spanned further east in the United States and Canada, but they have been extirpated from ~30% of their historical range (COSEWIC 2002; Fellows and Jones 2009). In Canada, curlews are listed as a species of “Special Concern” on Schedule 1 of the Species at Risk Act (Cannings 1999; Jones et al. 2008). In the United States, curlews are listed as a U.S. Fish and Wildlife Service Bird of Conservation Concern (Fellows and Jones 2009). The loss of short grass and mixed grass prairies (Wick et al. 2016), where much of North America's curlew population is found (COSEWIC 2002), has reduced the curlew's range (COSEWIC 2002; Fellows and Jones 2009) and will likely continue to impact their abundance and distribution.

Detection and count data from bird surveys are crucial for modeling and understanding population trends (e.g., Sauer et al. 2014; Smith and Edwards 2021; Sullivan et al. 2009), especially in light of anthropogenic change. Although structured surveys such as the Breeding Bird Survey can provide insight into long‐term trends (Sauer et al. 2014; Smith et al. 2019), especially at specific locations, these surveys can be limited by coverage (i.e., number and location of routes) as well as detection biases imposed by species' behavior (e.g., Ankori‐Karlinsky et al. 2022; Bianchini and Tozer 2023; Fellows and Jones 2009). Curlews are inconspicuous during nesting season, making them difficult to detect during this period (Fellows and Jones 2009). Nesting season coincides with when BBS routes are often completed, and therefore BBS surveys are largely unreliable for accurate curlew population estimates (Fellows and Jones 2009; Environment Canada 2012). As such, there is high value in community‐driven data collection, such as through eBird, and this approach can provide the opportunity to answer a wide range of environmental questions about conservation, species distribution, and more (Sullivan et al. 2009, 2014). eBird is a community science database in which users can submit bird observations in a standardized way (Sullivan et al. 2009), providing large‐scale spatial and temporal data that are useful for examining changes in a species' abundance, distribution, and range limits (Sullivan et al. 2009, 2014). However, eBird data is not without its limitations, including the veracity of the data, spatial or temporal bias, variation in observer effort, or bias in which species are reported (Dickinson et al. 2010; Johnston et al. 2021). As such, semi‐structured databases such as eBird provide excellent opportunities to examine large‐scale patterns and changes to distributions, but those must be interpreted within the limitations of the data source (La Sorte et al. 2018).

Curlews have a large range throughout North America, making it challenging to gain insight into their abundance and distribution using traditional survey methods. eBird is a potentially valuable resource for understanding these patterns. eBird data have previously been used to examine how species distributions and migration patterns have changed over time (e.g., Prytula et al. 2023; Sonnleitner et al. 2022). For example, Sonnleitner et al. (2022) found that the breeding season population centroids of western ( *Sialia mexicana* ), eastern ( *Sialia sialis* ), and mountain ( *Sialia currucoides* ) bluebirds have all shifted southward, whereas the migratory population centroids have shifted longitudinally toward the center of the continent. Similarly, a study on Vaux's ( *Chaetura vauxi* ) and chimney ( *Chaetura vauxi* ) swift using eBird data revealed that the breeding season population centroids of both species have shifted towards the center of the continent, a pattern potentially driven by urban encroachment and habitat loss along both coasts (Prytula et al. 2023). These studies illustrate the power of eBird to allow us to detect unexpected, and sometimes surprising, shifts in distributions that may go undetected through the use of traditional methods alone.

Although examining changes across an entire species range is important, understanding both large‐scale and regional distribution dynamics is critical for the effective management of local populations. Species with large ranges distributed across different eco‐regions will face different pressures from climate and land use (e.g., Conroy et al. 2012; Jones 2011; Pavlacky et al. 2017). As such, a more nuanced approach to examining the differences in each eco‐region, such as through the use of Bird Conservation Regions (BCRs), can be beneficial for understanding where populations may be the most vulnerable. The North American Bird Conservation Initiative Committee divided North America into 66 BCRs across Canada, the U.S., and Mexico as ecologically distinct regions that are defined by groupings of similar biotic communities (plants, wildlife) and abiotic characteristics (e.g., soil characteristics, climate) (Bird Studies Canada and NABCI 2014; CEC 1998). For resource management and conservation, this approach allows for improved domestic and international cooperation by working across relatively homogeneous habitat (Birds Studies Canada and NABCI 2014). Examining changes to the climate and habitat of the varying BCRs in which a species occurs can provide a better understanding of the species distribution and abundance patterns on a broad scale (Pavlacky et al. 2017) and may aid in the development of Strategic Habitat Management plans (e.g., Giocomo et al. 2009). The long‐billed curlew breeding range spans eight such BCRs, and given this expansive range and the variability in curlew population trends, the use of eBird is a powerful tool to fill the gap in our understanding of potential causes of both large‐scale and regional distribution shifts.

Here, we used eBird data to analyze curlew distribution dynamics across their entire North American range as well as within the BCRs that encompass their range. We predicted that curlews would show an overall northern range expansion within their North American range in response to warming temperatures at their northern range periphery. Furthermore, we predicted that curlew distributions would shift differently in response to the variable habitat loss and climatic stressors within each BCR. Specifically, we predicted that the Northern Rockies (BCR 10) and Prairie Potholes (BCR 11) would show a northern centroid shift due to warming in the northern periphery of these BCRs (Chaikowsky 2000; Wang et al. 2016 from ClimateBC). We also predicted that the Prairie Potholes (BCR 11), Badlands and Prairies (BCR 17), Shortgrass Prairie (BCR 18), and Central Mixed Grass Prairie (BCR 19) would show western centroid shifts resulting from high levels of grassland loss to agricultural land on the eastern edge of the Great Plains (Lark et al. 2020; Wick et al. 2016).

## 2 Methods

### 2.1 eBird Data

**FIGURE 1:**
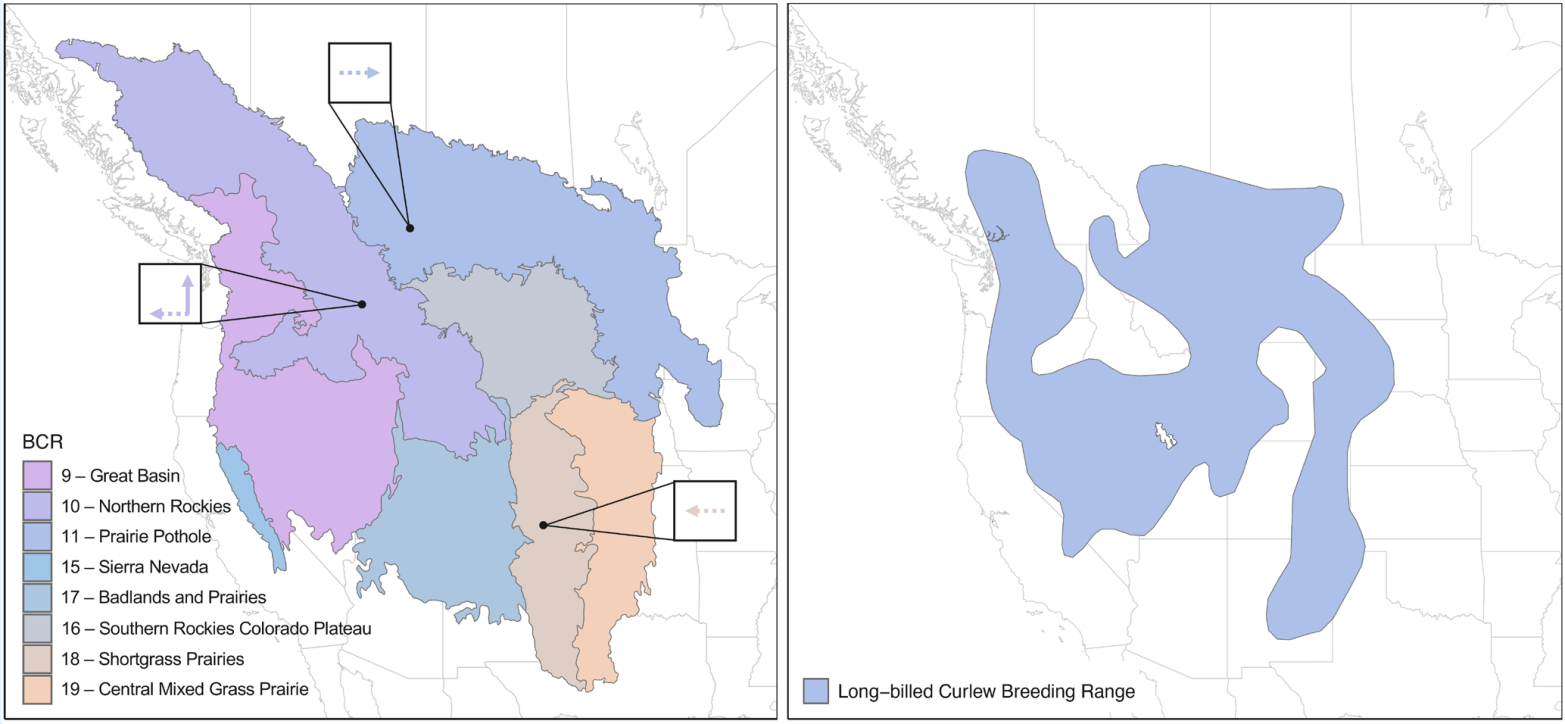
Left: Changes to the centroid locations of long‐billed curlews within Bird Conservation Regions (BCRs). The black dots represent the centroid position in 2022, and the arrows represent the direction of significant change. A dashed arrow represents *p* < 0.05 and a solid arrow represents *p* < 0.01. Right: Long‐billed curlew Breeding Range (breeding range data from BirdLife International and Handbook of the Birds of the World 2022).

Long‐billed curlew data from 2010 to 2022 were acquired from the community science database, eBird (eBird 2021). We began data collection in 2010, as data prior to that point are generally sparse and inadequate for our analysis. eBird provides checklists (single birding events) that include the species observed, the number of individuals per species, the location, the date and time, and the effort, measured by variables including the distance traveled during “traveling” observations, the length of time each checklist was recorded for, and the number of observers. We used the “auk” package (v0.6.0; Strimas‐Mackey, Miller, et al. 2023) in R (v4.3.1; R Core Team 2021) to explore and filter data and followed eBird Best Practices to filter the data (Strimas‐Mackey, Hochachka, et al. 2023). Specifically, we filtered the data to only include “stationary” or “traveling” protocols, omitting “incidental” and “historical” data. In addition, we removed traveling checklists that were > 5 km long and omitted checklists that lasted longer than 5 h to reduce variability in effort (Strimas‐Mackey, Hochachka, et al. 2023). We only included complete checklists, which refer to checklists in which all species seen or heard were recorded. This approach reduced the data from 264,661 checklists with an observation to 42,768 checklists. Our high number of exclusions highlights the importance of eBird contributors submitting complete checklists that contain all important data including the date, time, accurate geographic location, survey distance and duration, and the number of observers. The inclusion of these parameters greatly enhances the scientific merit and reliability of eBird data for research and analysis. Lastly, checklists that did not include curlew observations were zero‐filled, to provide non‐detection data. The resulting data were temporally restricted to May 1st to July 31st, which encompasses the long‐billed curlew breeding period (Dugger and Dugger 2020), and spatially restricted to within the Great Basin (BCR 9), Northern Rockies, Prairie Potholes, Sierra Nevada (BCR 15), Southern Rockies‐Colorado Plateau (BCR 16), Badlands and Prairies, and Central Mixed Grass Prairie, which encompasses the curlew breeding range within North America (Figure 1).

### 2.2 Breeding Range Limits and Centroid Positions

We created 10 km by 10 km grids across the long‐billed curlew breeding range and calculated the total number of complete checklists and checklists with curlew detections per year within each grid. We only retained grid cells with checklists in at least 5 years to reduce any spatial bias of grid cells that were poorly sampled. We chose to use 5 years, as that period encompassed roughly half the years in our study and balanced issues with using too few years (e.g., examining change over time with only 2–3 years of data) and stringency (e.g., data available in all years of the study), which would have greatly reduced our sample size and power to detect any patterns. We scored grid cells by whether a curlew was detected in each year. We calculated the centroid (latitude and longitude) of curlew detections each year for the entire range, as well as within each BCR using the “sf” R package (v 1.0‐12; Pebesma and Bivand 2023) to calculate the centroid and min/max latitude and longitude for all grid cells with an observation in a region for that year. The centroid was calculated by first unioning the grid cells when taking the centroid. The min/max latitude and longitude were taken from the bounding box around the grid cells. The centroid represents the geographic center of detections within each region. We also calculated the latitudinal and longitudinal bounds per year for the entire breeding range.

### 2.3 Statistical Analysis

We conducted a series of linear regressions in R (Version 4.3.1, R Core Team 2021) using the “lm” function to test for changes in the centroid position and range boundaries of curlews from 2010 to 2022 using position (latitude/longitude) as a response variable and year as a main effect. To control for the potential effect of changes in eBird checklist submissions over time, we also included the centroid position and range boundaries for all checklists, whether they included curlews or not, as a covariate in our analysis (all VIF < 10; mean VIF = 2.2 ± 1.9 SD). We converted the change in degrees after controlling for changes in eBird checklist submissions to kilometers. To account for the change in distance between longitudes towards the equator, we used the latitudinal location when calculating longitudinal changes. Linear regressions were calculated for the northern, eastern, southern, and western range limits, as well as the longitudinal and latitudinal centroid positions, for the entire breeding range. Within BCRs, we conducted linear regressions for the longitudinal and latitudinal centroid position. We calculated the distance of the estimated cumulative change (in kilometers) and yearly change (in degrees) in centroid positions and range limits using the slope of the linear regressions. Results are presented as mean ± standard error.

## 3 Results

### 3.1 Breeding Range Limits and Centroid Positions

#### 3.1.1 North American Breeding Range

Based on eBird data collected between 2010 and 2022 and after accounting for changes in eBird activity during that period, the northern range limit of curlews shifted north by 0.161 ± 0.054 °/year, for a cumulative change of ~233 km (*r* ^2^ = 0.40, *p* = 0.01). The western range limit expanded west by 0.183 ± 0.025 °/year (total change: ~194 km; adjusted *r* ^2^ = 0.27, *p* = 0.03). There were no changes to the southern range limit, eastern range limit, or centroid latitude or longitude positions (Table 1, all *p* > 0.05).

**TABLE 1:** Changes to centroid latitude and longitude of the long‐billed curlew breeding range from 2010 to 2022 within Bird Conservation Regions.

Bird Conservation Regions	*p*	Adjusted *R* ^2^	Estimated total distance and direction shifted
BCR 9—Great Basin (*n* = 5327)			
Centroid latitude	0.16	0.05	
Centroid longitude	0.18	0.02	
BCR 10—Northern Rockies (*n* = 2864)			
Centroid latitude	**0.008**	**0.44**	**84 km N**
Centroid longitude	**0.03**	**0.51**	**85 km W**
BCR 11—Prairie Pothole (*n* = 1409)			
Centroid latitude	0.50	0.38	
Centroid longitude	**0.01**	**0.56**	**77 km E**
BCR 15—Sierra Nevada (*n* = 183)			
Centroid latitude	0.53	0.10	
Centroid longitude	0.86	−0.192	
BCR 16—Southern Rockies Colorado Plateau (*n* = 218)			
Centroid latitude	0.49	0.20	
Centroid longitude	0.17	0.23	
BCR 17—Badlands and Prairies (*n* = 979)			
Centroid latitude	0.42	−0.001	
Centroid longitude	0.77	−0.124	
BCR 18—Shortgrass Prairie (*n* = 667)			
Centroid latitude	0.16	0.02	
Centroid longitude	**0.03**	**0.48**	**45 km W**
BCR 19—Central Mixed Grass Prairie (*n* = 97)			
Centroid latitude	0.38	0.07	
Centroid longitude	0.21	0.14	

*Note:* Bold values indicate *p* < 0.05. Sample size represents total number of checklists with curlews observed between 2010 and 2022.

#### 3.1.2 Bird Conservation Regions

After controlling for changes in checklist centroid over time, the centroid latitude position shifted north in the Northern Rockies by 0.084 ± 0.025 °/year, with a cumulative change of ~121 km (adjusted *r* ^2^ = 0.44, *p* = 0.008) between 2010 and 2022 (Table 1, Figure 1). The centroid longitudinal position shifted west in the Northern Rockies by 0.083 ± 0.025 °/year (total change: ~74 km; adjusted *r* ^2^ = 0.51, *p* = 0.03), east in the Prairie Potholes by 0.076 ± 0.025 °/year (total change: ~111 km; adjusted *r* ^2^ = 0.56, *p* = 0.008), and west in the Shortgrass Prairie by 0.064 ± 0.025 °/year (total change: ~85 km; adjusted *r* ^2^ = 0.48, *p* = 0.03). We detected no changes to the centroid latitude or longitude in the Great Basin, Sierra Nevada, Southern Rockies, Colorado Plateau, Badlands and Prairies, or Central Mixed Grass Prairie (all *p* > 0.16).

## 4 Discussion

Using eBird data spanning 13 years, we analyzed relative changes to the North American distribution of long‐billed curlews, as well as changes within the eight BCRs that overlap the curlew's breeding range. Our results indicate that across the entirety of their breeding range, curlews have expanded their northern range limit—a pattern consistent with northern range expansions detected across taxa in response to warming temperatures resulting from climate change (Parmesan and Yohe 2003). When we looked at shifts in range centroids within BCRs, we found high variability in the direction and magnitude of changes, indicating that warming temperatures may have different effects within different bioclimatic zones represented by BCRs and that other factors, such as spatial differences in habitat loss across BCRs, may be driving patterns of distributional change at a regional scale.

Our results indicate that curlews expanded their northern breeding range limit by approximately 198 km over the last 13 years. Although curlews have expanded their northern range overall, they have not contracted their southern range, suggesting a range expansion rather than a range shift. Furthermore, we also detected northward movement of the centroid latitude position within the Northern Rockies (~84 km). Warmer temperatures in the north allow for an earlier onset of spring and may create new climatically suitable regions for species to move into (Fraser 1999; Skagen and Adams 2012; Jarzyna et al. 2016; Nixon et al. 2016). Northern range expansions and changes in species distribution in response to a warming climate are becoming increasingly common among bird populations (Chen et al. 2011; Hitch and Leberg 2007; Nixon et al. 2016; Rushing et al. 2020). However, for these range shifts to occur there must be suitable habitat for the species to move into (e.g., Nixon et al. 2016). Curlew habitat in the Northern Rockies is composed mainly of agricultural land, which has increased in this region in recent years and may be providing habitat to facilitate this northern range expansion (Unpublished data), though the productivity and suitability of these sites remain unknown. As such, the northern range expansion and centroid shift in North America, as well as the northern centroid shift in the Northern Rockies, are likely due to the combined effects of agricultural land conversion and warming temperatures that have made previously unsuitable habitat now available.

The other BCR that comprises the northern extent of the curlew range—the Prairie Potholes—showed an eastern centroid shift. Breeding Bird Survey data showed a significant long‐term (1970–2022) and short‐term (2011–2022) negative trend of curlew abundance within the Canadian portion of the Prairie Potholes (Smith et al. 2019). This population decline of curlews within the Prairie Potholes may explain the observed eastern centroid shift, as much of the northwestern portion of this BCR falls within Canada. Thus, if the Canadian portion is declining, the centroid would naturally shift towards the southeastern section of this BCR. Curlews were previously extirpated from around 30% of their easternmost historical range (COSEWIC 2002; Fellows and Jones 2009), including the eastern regions of North and South Dakota. In recent years, agricultural conversion in the Dakotas has intensified (Lark et al. 2020), and if curlews are now potentially occupying agricultural lands in these regions, as they may be in north central British Columbia, the eastern centroid movement in the Prairie Potholes may represent a recolonization of their previously lost range. Much of the remaining grasslands of the Great Plains are now highly fragmented or have woody encroachment (Wick et al. 2016), making this habitat unsuitable for curlews (Cannings 1999). The conversion of these unsuitable regions of grassland habitats to agriculture may provide curlews with habitat that appears to be of high quality. Occupation of agricultural habitat will likely be a short‐term benefit to curlews as agricultural lands may act as population sinks (e.g., Bollinger et al. 1990; Green et al. 1997; Perlut et al. 2008).

In addition to a northern range expansion, we also observed a change to the western range limit of the long‐billed curlew breeding range—a pattern that is driven by shifts in the Northern Rockies, given that it is the westernmost BCR. The newly climatically suitable northern region that curlews appear to have recently shifted into is northwest of their previously known breeding range. Therefore, this western range expansion is likely linked to the northern range expansion. In addition, we observed western centroid shifts in the Southern Rockies, Colorado Plateau, and the Shortgrass Prairie. Although the western centroid shift in the Northern Rockies is hypothesized to be linked to climate change and habitat gain through agricultural expansion, the western centroid shift in the Shortgrass Prairie may be related to localized habitat loss within this region. The Shortgrass Prairie BCR is characterized by arid environments with shortgrass prairies as the dominant ecosystem (Bird Studies Canada and NABCI 2014). Shortgrass prairies in the mid and southwestern United States are facing heavy degradation and conversion by agricultural activities and urban development (Comer et al. 2018). These losses occur largely on the eastern limit of the curlews breeding range in the Great Plains and may be driving the observed western shift in curlew centroid distribution within this BCR.

Climate change and habitat loss continue to be omnipresent threats, influencing the distribution of species and threatening their persistence (Jaureguiberry et al. 2022; Mantyka‐Pringle et al. 2012). Future studies should aim to investigate changes in habitat and climate within each BCR to understand the threats curlews are facing. An estimated 62% of grasslands in North America have been lost (Comer et al. 2018). Habitat loss can influence a species' distribution directly and indirectly. Directly, losing large amounts of habitat may change a species' distribution as they shift to find suitable habitats. Indirectly, agricultural lands that are replacing grassland ecosystems may act as population sinks (e.g., Bollinger et al. 1990; Green et al. 1997; Perlut et al. 2008), ultimately leading to population declines, which in turn would influence a population's centroid position through effects on local abundance.

In terms of conservation implications, the observed shifts in breeding distribution highlight the dynamic responses of long‐billed curlews to changing environmental conditions such as climate change and habitat loss. Curlew productivity, however, remains largely unknown in these novel breeding areas (COSEWIC 2002), and factors related to human activity will likely control the likelihood of long‐term establishment. Agricultural lands present a complex set of anthropogenic threats that differ from those in undisturbed habitats such as grasslands. During the curlew nesting season, agricultural lands are subject to regular mowing (Tews et al. 2013; Stanton et al. 2018), which can lead to mortality (e.g., COSEWIC 2002; Green et al. 1997; Perlut et al. 2008), nest abandonment (e.g., Bollinger et al. 1990) and destruction (e.g., Green et al. 1997; Perlut et al. 2008; Kentie et al. 2015), and increased predation rates (e.g., COSEWIC 2002; Bollinger et al. 1990; Beja et al. 2013). Additionally, the increased use of pesticides associated with agricultural intensification can lead to reduced food availability and mortality from exposure (Boatman et al. 2004; Stanton et al. 2018). These threats mean agricultural lands may act as population sinks (e.g., Green et al. 1997; Perlut et al. 2008), ultimately reducing the long‐term viability of curlews in this newly expanded range. Future work on curlew productivity in this newly expanded range will be crucial in understanding and informing effective management of curlews.

Although it appears long‐billed curlew range dynamics on a large scale may be influenced by climate change, different patterns of distribution shifts at the BCR level indicate other factors, such as habitat availability, may be influencing local distribution and density, and may interact with changes brought by climate change. Our work demonstrates the importance of examining changes to distribution patterns at both regional and range‐wide scales and highlights the utility of community science data to better predict how future climate change and habitat loss scenarios may impact vulnerable species.

## Author Contributions

**Kelsey Freitag:** conceptualization (equal), formal analysis (equal), funding acquisition (equal), investigation (lead), methodology (equal), visualization (lead), writing – original draft (lead), writing – review and editing (lead). **Ann E. McKellar:** conceptualization (equal), investigation (equal), methodology (equal), project administration (equal), supervision (equal), writing – review and editing (equal). **David W. Bradley:** conceptualization (equal), writing – review and editing (equal). **Scott A. Flemming:** conceptualization (equal), writing – review and editing (equal). **Steffi LaZerte:** conceptualization (equal), data curation (lead), formal analysis (equal), methodology (equal), validation (lead), writing – review and editing (equal). **Mateen Shaikh:** conceptualization (equal), formal analysis (equal), methodology (equal), writing – review and editing (equal). **Matthew W. Reudink:** conceptualization (equal), formal analysis (equal), funding acquisition (equal), investigation (equal), methodology (equal), project administration (equal), resources (equal), supervision (equal), writing – review and editing (equal).

## Conflicts of Interest

The authors declare no conflicts of interest.

## Data Availability

Data is available and can be found on Dryad at https://doi.org/10.5061/dryad.w3r22813c. Code is available and can be found on Zenodo at https://doi.org/10.5281/zenodo.15320130.

## References

[ece371537-bib-0001] Ankori‐Karlinsky, R. , M. Kalyuzhny , K. F. Barnes , et al. 2022. “North American Breeding Bird Survey Underestimates Regional Bird Richness Compared to Breeding Bird Atlases.” Ecosphere 13, no. 2: e3925. 10.1002/ecs2.3925.

[ece371537-bib-0062] Beja, P. , S. Schindler , J. Santana , et al. 2013. “Predators and Livestock Reduce Bird Nest Survival in Intensive Mediterranean Farmland.” European Journal of Wildlife Research 60, no. 2: 249–258. 10.1007/s10344-013-0773-0.

[ece371537-bib-0002] Bianchini, K. , and D. Tozer . 2023. “Using Breeding Bird Survey and eBird Data to Improve Marsh Bird Monitoring Abundance Indices and Trends.” Avian Conservation and Ecology 18, no. 1: art4. 10.5751/ACE-02357-180104.

[ece371537-bib-0003] Bird Studies Canada and NABCI . 2014. Bird Conservation Regions. Bird Studies Canada on behalf of the North American Bird Conservation Initiative. https://birdscanada.org/bird‐science/nabci‐bird‐conservation‐regions.

[ece371537-bib-0004] BirdLife International and Handbook of the Birds of the World . 2022. “Bird Species Distribution Maps of the World. Version 2022.2.” http://datazone.birdlife.org/species/requestdis.

[ece371537-bib-0005] Birds Canada and Environment and Climate Change Canada . 2024. The State of Canada's Birds Report. NatureCounts. 10.71842/8bab-ks08.

[ece371537-bib-0063] Boatman, N. D. , N. W. Brickle , J. D. Hart , et al. 2004. “Evidence for the Indirect Effects of Pesticides on Farmland Birds.” Ibis 146, no. s2: 131–143. 10.1111/j.1474-919X.2004.00347.x.

[ece371537-bib-0006] Bollinger, E. K. , P. B. Bollinger , and T. A. Gavin . 1990. “Effects of Hay‐Cropping on Eastern Populations of the Bobolink.” Wildlife Society Bulletin 18, no. 2: 142–150.

[ece371537-bib-0007] Cannings, R. J. 1999. Status of the Long‐Billed Curlew in British Columbia. Wildlife Working Report, No. WR‐96. Ministry of Environment, Lands, and Parks, Wildlife Branch.

[ece371537-bib-0008] CEC (Commission for Environmental Cooperation) . 1998. A Proposed Framework for Delineating Ecologically‐Based Planning, Implementation, and Evaluation Units for Cooperative Bird Conservation in the U.S. U.S. Fish and Wildlife Service. https://www.partnersinflight.org/wpcontent/uploads/2019/01/CEC‐Proposed‐BCR‐Framework1998.pdf.

[ece371537-bib-0009] Chaikowsky, C. L. A. 2000. Analysis of Alberta Temperature Observations and Estimates by Global Climate Models. Branch.

[ece371537-bib-0010] Chen, I.‐C. , J. K. Hill , R. Ohlemüller , D. B. Roy , and C. D. Thomas . 2011. “Rapid Range Shifts of Species Associated With High Levels of Climate Warming.” Science 333, no. 6045: 1024–1026. 10.1126/science.1206432. 21852500

[ece371537-bib-0011] Comer, P. J. , J. C. Hak , K. Kindscher , E. Muldavin , and J. Singhurst . 2018. “Continent‐Scale Landscape Conservation Design for Temperate Grasslands of the Great Plains and Chihuahuan Desert.” Natural Areas Journal 38, no. 2: 196–211. 10.3375/043.038.0209.

[ece371537-bib-0012] Conroy, M. J. , K. W. Stodola , and R. J. Cooper . 2012. “Effective Use of Data From Monitoring Programs and Field Studies for Conservation Decision Making: Predictions, Designs and Models Working Together.” Journal of Ornithology 152, no. S2: 325–338. 10.1007/s10336-011-0687-0.

[ece371537-bib-0013] COSEWIC . 2002. OSEWIC Assessment and Status Report on the Long‐Billed Curlew *Numenius americanus* in Canada. Committee on the Status of Endangered Wildlife in Canada. Vii +31.

[ece371537-bib-0014] Dickinson, J. L. , B. Zuckerberg , and D. N. Bonter . 2010. “Citizen Science as an Ecological Research Tool: Challenges and Benefits.” Annual Review of Ecology, Evolution, and Systematics 41: 149–172.

[ece371537-bib-0015] Dugger, B. D. , and K. M. Dugger . 2020. Long‐Billed Curlew (Numenius Americanus), Version 1.0. Birds of the World. Cornell Lab of Ornithology. 10.2173/bow.lobcur.01.

[ece371537-bib-0016] eBird . 2021. eBird: An Online Database of Bird Distribution and Abundance [Web Application]. eBird, Cornell Lab of Ornithology. http://www.ebird.org.

[ece371537-bib-0017] Environment Canada . 2012. Management Plan for the Long‐Billed Curlew (Numenius americanus) in Canada [Proposed]. Species at Risk Act Management Plan Series. Environment Canada. iii +24.

[ece371537-bib-0018] Fellows, S. D. , and S. L. Jones . 2009. Status Assessment and Conservation Action Plan for the Long‐Billed Curlew (Numenius americanus). US Department of Interior Fish and Wildlife Service. Biological Technical Publications, FWS/BTP‐R6012‐2009.

[ece371537-bib-0019] Fraser, D. F. 1999. “Species at the Edge: The Case for Listing of ‘Peripheral’ Species.”

[ece371537-bib-0021] Giocomo, J. J. , D. A. Buehler , and J. Fitzgerald . 2009. “Integrating Grassland and Shrubland Bird Conservation With the Northern Bobwhite Conservation Initiative for the Central Hardwoods Bird Conservation Region.” In Proceedings of the Fourth International Partners in Flight Conference: Tundra to Tropics, 545–556. Central Hardwoods Joint Venture.

[ece371537-bib-0022] Green, R. E. , G. A. Tyler , T. J. Stowe , and A. V. Newton . 1997. “A Simulation Model of the Effect of Mowing of Agricultural Grassland on the Breeding Success of the Corncrake ( *Crex Crex* ).” Journal of Zoology 243, no. 1: 81–115. 10.1111/j.1469-7998.1997.tb05758.x.

[ece371537-bib-0023] Hitch, A. T. , and P. L. Leberg . 2007. “Breeding Distributions of North American Bird Species Moving North as a Result of Climate Change.” Conservation Biology 21, no. 2: 534–539. 10.1111/j.1523-1739.2006.00609.x. 17391203

[ece371537-bib-0024] Jarzyna, M. A. , B. Zuckerberg , A. O. Finley , and W. F. Porter . 2016. “Synergistic Effects of Climate and Land Cover: Grassland Birds Are More Vulnerable to Climate Change.” Landscape Ecology 31, no. 10: 2275–2290. 10.1007/s10980-016-0399-1.

[ece371537-bib-0025] Jaureguiberry, P. , N. Titeux , M. Wiemers , et al. 2022. “The Direct Drivers of Recent Global Anthropogenic Biodiversity Loss.” Science Advances 8, no. 45: eabm9982. 10.1126/sciadv.abm9982. 36351024 PMC9645725

[ece371537-bib-0026] Johnston, A. , W. M. Hochachka , M. E. Strimas‐Mackey , et al. 2021. “Analytical Guidelines to Increase the Value of Community Science Data: An Example Using eBird Data to Estimate Species Distributions.” Diversity and Distributions 27, no. 7: 1265–1277.

[ece371537-bib-0027] Jones, J. P. G. 2011. “Monitoring Species Abundance and Distribution at the Landscape Scale.” Journal of Applied Ecology 48, no. 1: 9–13. 10.1111/j.1365-2664.2010.01917.x.

[ece371537-bib-0028] Jones, S. L. , C. S. Nations , S. D. Fellows , and L. L. McDonald . 2008. “Breeding Abundance and Distribution of Long‐Billed Curlews (*Numenius Americanus*) in North America.” Waterbirds 31, no. 1: 1–14.

[ece371537-bib-0061] Kentie, R. , C. Both , J. C. E. W. Hooijmeijer , and T. Piersma . 2015. “Management of Modern Agricultural Landscapes Increases Nest Predation Rates in Black‐Tailed Godwits *Limosa limosa* .” Ibis 157, no. 3: 614–625. 10.1111/ibi.12273.

[ece371537-bib-0029] La Sorte, F. A. , and W. Jetz . 2012. “Tracking of Climatic Niche Boundaries Under Recent Climate Change.” Journal of Animal Ecology 81: 914–925. 22372840 10.1111/j.1365-2656.2012.01958.x

[ece371537-bib-0030] La Sorte, F. A. , C. A. Lepczyk , J. L. Burnett , A. H. Hurlbert , M. W. Tingley , and B. Zuckerberg . 2018. “Opportunities and Challenges for Big Data Ornithology.” Condor 120: 414–426.

[ece371537-bib-0031] Lark, T. J. , S. A. Spawn , M. Bougie , and H. K. Gibbs . 2020. “Cropland Expansion in the United States Produces Marginal Yields at High Costs to Wildlife.” Nature Communications 11, no. 1: 4295. 10.1038/s41467-020-18045-z. PMC7481238 32908130

[ece371537-bib-0032] Mac Nally, R. , A. F. Bennett , J. R. Thomson , et al. 2009. “Collapse of an Avifauna: Climate Change Appears to Exacerbate Habitat Loss and Degradation.” Diversity and Distributions 15, no. 4: 720–730. 10.1111/j.1472-4642.2009.00578.x.

[ece371537-bib-0033] Mantyka‐Pringle, C. S. , T. G. Martin , and J. R. Rhodes . 2012. “Interactions Between Climate and Habitat Loss Effects on Biodiversity: A Systematic Review and Meta‐Analysis.” Global Change Biology 18, no. 4: 1239–1252. 10.1111/j.1365-2486.2011.02593.x.

[ece371537-bib-0034] Mantyka‐Pringle, C. S. , P. Visconti , M. Di Marco , T. G. Martin , C. Rondinini , and J. R. Rhodes . 2015. “Climate Change Modifies Risk of Global Biodiversity Loss due to Land‐Cover Change.” Biological Conservation 187: 103–111. 10.1016/j.biocon.2015.04.016.

[ece371537-bib-0035] Nixon, A. E. , R. J. Fisher , D. Stralberg , E. M. Bayne , and D. R. Farr . 2016. “Projected Responses of North American Grassland Songbirds to Climate Change and Habitat Availability at Their Northern Range Limits in Alberta, Canada.” Avian Conservation and Ecology 11, no. 2: art2. 10.5751/ACE-00866-110202.

[ece371537-bib-0036] Parmesan, C. , and G. Yohe . 2003. “A Globally Coherent Fingerprint of Climate Change Impacts Across Natural Systems.” Nature 421, no. 6918: 37–42. 10.1038/nature01286. 12511946

[ece371537-bib-0037] Pavlacky, D. C. , P. M. Lukacs , J. A. Blakesley , et al. 2017. “A Statistically Rigorous Sampling Design to Integrate Avian Monitoring and Management Within Bird Conservation Regions Christian Andrew Hagen.” PLoS One 12, no. 10: e0185924. 10.1371/journal.pone.0185924. 29065128 PMC5655431

[ece371537-bib-0038] Pebesma, E. , and R. Bivand . 2023. Spatial Data Science: With Applications in R. Chapman and Hall/CRC. 10.1201/9780429459016.

[ece371537-bib-0039] Perlut, N. G. , A. M. Strong , T. M. Donovan , and N. J. Buckley . 2008. “Grassland Songbird Survival and Recruitment in Agricultural Landscapes: Implications for Source–Sink Demography.” Ecology 89, no. 7: 1941–1952. 10.1890/07-0900.1. 18705380

[ece371537-bib-0040] Prytula, E. , M. Reudink , S. LaZerte , J. Sonnleitner , and A. McKellar . 2023. “Shifts in Breeding Distribution, Migration Timing, and Migration Routes of Two North American Swift Species.” Journal of Field Ornithology 94, no. 3: art14. 10.5751/JFO-00341-940314.

[ece371537-bib-0041] R Core Team . 2021. R: A Language and Environment for Statistical Computing. R Foundation for Statistical Computing. https://www.R‐project.org/.

[ece371537-bib-0042] Rosenberg, K. V. , A. M. Dokter , P. J. Blancher , et al. 2019. “Decline of the North American Avifauna.” Science 366, no. 6461: 120–124. 10.1126/science.aaw1313. 31604313

[ece371537-bib-0043] Rubenstein, M. A. , S. R. Weiskopf , R. Bertrand , et al. 2023. “Climate Change and the Global Redistribution of Biodiversity: Substantial Variation in Empirical Support for Expected Range Shifts.” Environmental Evidence 12: 7. 39294691 10.1186/s13750-023-00296-0 PMC11378804

[ece371537-bib-0044] Rushing, C. S. , J. A. Royle , D. J. Ziolkowski , and K. L. Pardieck . 2020. “Migratory Behavior and Winter Geography Drive Differential Range Shifts of Eastern Birds in Response to Recent Climate Change.” Proceedings of the National Academy of Sciences of the United States of America 117, no. 23: 12897–12903. 10.1073/pnas.2000299117. 32457137 PMC7293646

[ece371537-bib-0045] Sauer, J. R. , J. E. Hines , and J. Fallon . 2001. The North American Breeding Bird Survey, Results and Analysis 1966–2000. Version 2001.2. USGS Patuxent Wildlife Research Center.

[ece371537-bib-0046] Sauer, J. R. , J. E. Hines , J. Fallon , K. L. Pardieck , D. J. Ziolkowski , and W. A. Link . 2014. The North American Breeding Bird Survey, Results and Analysis 1966–2013, Version 01.30.2015. USGS Patuxent Wildlife Research Center.

[ece371537-bib-0047] Skagen, S. K. , and A. A. Y. Adams . 2012. “Weather Effects on Avian Breeding Performance and Implications of Climate Change.” Ecological Applications 22, no. 4: 1131–1145. 10.1890/11-0291.1. 22827123

[ece371537-bib-0048] Smith, A. C. , M.‐A. R. Hudson , V. I. Aponte , W. B. English , and C. M. Francis . 2024. North American Breeding Bird Survey—Canadian Trends Website, Data‐Version 2023. Environment and Climate Change Canada.

[ece371537-bib-0049] Smith, A. C. , and B. P. M. Edwards . 2021. “North American Breeding Bird Survey Status and Trend Estimates to Inform a Wide Range of Conservation Needs, Using a Flexible Bayesian Hierarchical Generalized Additive Model.” Condor 123, no. 1: duaa065. 10.1093/ornithapp/duaa065.

[ece371537-bib-0050] Smith, A. C. , M.‐A. R. Hudson , V. Aponte , and C. M. Francis . 2019. North American Breeding Bird Survey–Canadian Trends Website, Data‐Version 2017. Environment and Climate Change Canada.

[ece371537-bib-0051] Sonnleitner, J. , S. LaZerte , A. E. McKellar , N. J. Flood , and M. Reudink . 2022. “Rapid Shifts in Migration Routes and Breeding Latitude in North American Bluebirds.” Ecosphere 13, no. 12: e4316.

[ece371537-bib-0052] Stanton, R. L. , C. A. Morrissey , and R. G. Clark . 2018. “Analysis of Trends and Agricultural Drivers of Farmland Bird Declines in North America: A Review.” Agriculture, Ecosystems & Environment 254: 244–254. 10.1016/j.agee.2017.11.028.

[ece371537-bib-0053] Strimas‐Mackey, M. , W. M. Hochachka , V. Ruiz‐Gutierrez , et al. 2023. Best Practices for Using eBird Data. Version 2.0. Cornell Lab of Ornithology. 10.5281/zenodo.3620739.

[ece371537-bib-0054] Strimas‐Mackey, M. , E. T. Miller , and W. Hochachka . 2023. “auk: eBird Data Extraction and Processing With AWK. R Package Version 0.7.0.” https://cornelllabofornithology.github.io/auk/.

[ece371537-bib-0055] Sullivan, B. L. , J. L. Aycrigg , J. H. Barry , et al. 2014. “The eBird Enterprise: An Integrated Approach to Development and Application of Citizen Science.” Biological Conservation 169: 31–40. 10.1016/j.biocon.2013.11.003.

[ece371537-bib-0056] Sullivan, B. L. , C. L. Wood , M. J. Iliff , R. E. Bonney , D. Fink , and S. Kelling . 2009. “eBird: A Citizen‐Based Bird Observation Network in the Biological Sciences.” Biological Conservation 142, no. 10: 2282–2292. 10.1016/j.biocon.2009.05.006.

[ece371537-bib-0060] Tews, J. , D. G. Bert , and P. Mineau . 2013. “Estimated Mortality of Selected Migratory Bird Species from Mowing and Other Mechanical Operations in Canadian Agriculture.” Avian Conservation and Ecology 8, no. 2: 8. 10.5751/ace-00559-080208.

[ece371537-bib-0057] Vickery, P. D. , J. R. Herkert , F. L. Knopf , J. Ruth , and C. E. Keller . 2000. “Grassland Birds: An Overview of Threats and Recommended Management Strategies.” In Strategies for Bird Conservation: The Partners in Flight Planning Process; Proceedings of the 3rd Partners in Flight Workshop; 1995 October 1–5, 74–77. U.S. Forest Service, Rocky Mountain Research Station.

[ece371537-bib-0058] Wang, T. , A. Hamann , D. Spittlehouse , and C. Carroll . 2016. “Locally Downscaled and Spatially Customizable Climate Data for Historical and Future Periods for North America.” PLoS One 11, no. 6: e0156720. 10.1371/journal.pone.0156720. 27275583 PMC4898765

[ece371537-bib-0059] Wick, A. F. , B. A. Geaumont , K. K. Sedivec , and J. R. Hendrickson . 2016. “Grassland Degradation.” In Biological and Environmental Hazards, Risks, and Disasters, 257–276. Elsevier. 10.1016/B978-0-12-394847-2.00016-4.

